# Effect of Convective Cooling on the Temperature in a Friction System with Functionally Graded Strip

**DOI:** 10.3390/ma16155228

**Published:** 2023-07-25

**Authors:** Aleksander Yevtushenko, Michał Kuciej, Katarzyna Topczewska, Przemysław Zamojski

**Affiliations:** Faculty of Mechanical Engineering, Bialystok University of Technology (BUT), 45C Wiejska Street, 15-351 Bialystok, Poland; a.yevtushenko@pb.edu.pl (A.Y.); m.kuciej@pb.edu.pl (M.K.); p.zamojski@pb.edu.pl (P.Z.)

**Keywords:** frictional heating, convective cooling, temperature, functionally graded material

## Abstract

An exact solution of the boundary-value problem of heat conduction was obtained with consideration of heat generation due to friction and convective cooling for the strip/semi-space system. Analytical solutions to this problem are known for the case with both friction elements made of homogeneous materials or a composite layer with a micro-periodic structure. However, in this study, the strip is made of a two-component functionally gradient material (FGM). In addition, the exact, asymptotic solutions were also determined at small and large values of the Fourier number. By means of Duhamel’s theorem, it was shown that the developed solution for a constant friction power allows to obtain appropriate solutions with a changing time profile of this value during heating. Numerical analysis in dimensionless form was carried out for the FGM (ZrO_2_—Ti-6Al-4V) strip in combination with the cast iron semi-space. The influence of the convective cooling intensity (Biot number) on the temperature field in the considered friction system was investigated. The developed mathematical model allows for a quick estimation of the maximum temperature of systems, in which one of the elements (FGM strip) is heated on the friction surface and cooled by convection on the free surface.

## 1. Introduction

Analytical models of the frictional heating process in the pad/disc system are based on the experimentally confirmed assumption that the main part of the heat generated by friction is absorbed inside both sliding elements in the direction perpendicular to the contact surface [1,2,3]. This makes it possible to ignore changes in the temperature gradient in the other two directions, parallel to this surface [4]. As a consequence, the temperature field of the friction element is determined based on an analytical or analytical–numerical solution of the one-dimensional boundary-value problem of heat conduction for a two-element semi-space/semi-space, strip/semi-space and strip/strip systems, made of homogeneous materials [5,6]. The choice of one of these geometric schemes depends on the size of the effective depth of heat penetration $d_{eff}$ to the insides of each element of the friction pair [7]. Various formulas are used to estimate this parameter at the design stage, and one of the most commonly used is $d_{eff}=\sqrt{3kt_{s}}$, where k is the coefficient of the thermal diffusivity of the considered element and $t_{s}$ is the time of the frictional heating process [8]. If it turns out that $d_{eff}<d$, where d is the thickness of the element (pad, disc, etc.), during the formulation of the thermal problem of friction, then this element was considered as a semi-bounded body (half-space); otherwise, it was modeled with a strip.

In this article, the strip/semi-space friction system is considered, so we focused on the most important results directly related to it. First, the process of heat generation during braking with a constant deceleration under perfect thermal conditions of friction between the layer and the half-space was considered. During the entire heating process, the free surface of the strip was maintained at a constant (initial) temperature, or it was adiabatic (thermally insulated) [9]. The exact solutions of the corresponding boundary-value thermal problems of friction formulated in this way were obtained by the superposition method using the mathematical apparatus of the Laplace integral transform. Calculations were made for a metal–ceramic (FMC-11) layer and a cast iron (ChNMKh) half-space.

The solutions obtained in article [9] were generalized by taking into consideration the heat exchange with the surrounding environment on the free surface of the strip, according to Newton’s law [10]. The transition from the space of transforms to the space of originals was made using the integration technique in the plane of the complex parameter of the Laplace transform. Numerical analysis was carried out for the steel strip and semi-space made of aluminum. Using the Biot number, the influence of the intensity of convective cooling of the free surface of the steel strip during its sliding with a constant velocity on the surface of the aluminum semi-space was investigated.

The next studies concerned the modeling of imperfect thermal contact between the strip and the semi-space [11,12]. Such contact takes place in most cases during sliding and accompanying frictional heating of rough rubbing surfaces [13,14]. They assume that the resulting temperature jump on the contact surface is proportional to the differences in the intensity of heat fluxes directed along the normal from this surface to the insides of the sliding bodies. The coefficient of proportionality is the coefficient of the contact heat transfer—a parameter inversely proportional to the thermal resistance of the contact surface. An exact solution of the thermal problem of friction was obtained with uniform sliding of the strip over the surface of the semi-space, taking into account the imperfect thermal contact and the preservation of the initial temperature on the free surface of the strip [11]. The subsequent numerical analysis for the layer made of FMC-11 and the cast iron (ChNMKh) half-space made it possible, in particular, to determine the applicability ranges of the conditions of the perfect and imperfect thermal contact of friction for the considered pair. Another analytical model was developed to simulate the temperature field of the pad/disc system during braking with a constant deceleration under constant contact pressure [12] based on the strip/semi-space scheme. This model takes into consideration the thermal resistance of the contact surface with simultaneous convective cooling on the free surface of the strip.

It should be noted that all the mentioned works concerned the case of homogeneous materials of both elements of the friction couple. However, nowadays with the frictional elements of modern tribosystems, the nonhomogeneous materials are more likely to be used such as functionally graded materials (FGMs) [15,16,17]. Their usage is attractive to engineers and material scientists given the continuous grading and tailoring capabilities, when compared to traditional monolithic counterparts [18,19]. Usually, functionally graded friction materials are two-component composites made of ceramic and metal. So, the friction elements have high heat and wear resistance of ceramic on the outer zone, as well as the mechanical strength of the metal inside elements at the same time [20,21]. A smooth transition of composition from the outer surface to the inside ensure a reduction in stress concentration and an increase in fracture strength [21,22]. Research shows that controlling the gradient parameter of FGM can reduce residual tensile stress and improve thermal shock resistance [23]. Many computational models have been recently developed to evaluate the thermal response of FGMs, which is crucial for predicting failure mechanisms and designing friction couples [24,25,26,27,28,29,30,31,32,33]. Comprehensive reviews of the literature on thermal and thermoelastic problems of friction for functionally graded materials are given in our previous articles [34,35,36]. In these studies, the authors obtained the solutions of the boundary-value problems of heat conduction for a coating (layer) made of FGM with a heated surface that was ideally thermally connected to the surface of a homogeneous substrate (half-space) [35]. Then, the process of heat generation in a friction system consisting of a homogeneous semi-space sliding on the surface of the FGM strip applied to a homogeneous semi-space was investigated [36]. In both of these articles, the FGM layer performed the role of the thermal-barrier coating (TBC), allowing for more efficient heat dissipation from the heated surface. However, in this article, the FGM strip is one of the elements of the friction pair, absorbing the heat generated as a result of friction on the contact surface and cooled by convection on the free surface. The latter factor has not been taken into consideration in analytical models of friction heating involving FGM so far.

## 2. Statement to the Problem

**Figure 1:**
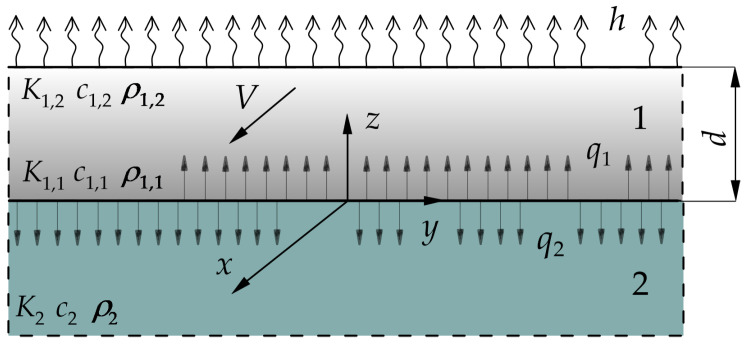
Scheme of the friction system strip/semi-space.

Let the strip 0≤z≤d at the initial moment t=0 begin to slide with constant velocity $V_{0}$ in the positive direction of the axis Ox on the outer surface of the semi-space z≤0 (Figure 1). The strip is made of a two-component functionally graded material, which has exponentially increasing thermal conductivity coefficient along the thickness, whereas the material of semi-space is homogeneous. Due to the friction on the contact surface z=0, the heat is generated and absorbed by each body of the friction couple. It was assumed that the thermal contact of friction between the strip and the semi-space is full. This means that the temperature of the friction surfaces of the strip and the half-space is the same, and the sum of the intensities of heat fluxes directed along the normal from the contact surface to the insides of each body is equal to the specific friction power $q_{0}=fp_{0}V_{0}$, where f is the coefficient of friction, and $p_{0}$ is the contact pressure. The free surface of the strip z=d is convectively cooled with a constant coefficient of heat transfer h. The initial temperature $T_{0}$ of the considered system is constant. A more detailed description of the assumptions of the mathematical model is given in our previous article [34].

The aim of this study is to explain the effect of FGM on the transient temperature fields T(z,t), −∞<z≤d, t≥0 of the strip and the semi-space. For this purpose, based on the above assumptions, the following thermal problem of friction for a single braking process in relation to the temperature rise $\Theta (z,t)=T(z,t)-T_{0}$ was formulated:

(1) $$\frac{\partial }{\partial z}\left[K_{1}(z)\frac{\partial \Theta (z,t)}{\partial z}\right]-\rho_{1}c_{1}\frac{\partial \Theta (z,t)}{\partial t}=0,\;0<z<d,\;t>0,$$

(2) $$K_{2}\frac{\partial^{2}\Theta (z,t)}{\partial z^{2}}-\rho_{2}c_{2}\frac{\partial \Theta (z,t)}{\partial t}=0,\;z<0,\;t>0,$$

(3) $$\Theta (0^{+},t)=\Theta (0^{-},t),\;t>0,$$

(4) $${{K_{2}\frac{\partial \Theta (z,t)}{\partial z}|}_{z=0^{-}}-K_{1}(z)\frac{\partial \Theta (z,t)}{\partial z}|}_{z=0^{+}}=q_{0},\;t>0,$$

(5) $${K_{1}(z)\frac{\partial \Theta (z,t)}{\partial z}|}_{z=d}=h[T_{0}-T(d,t)],\;t>0,$$

(6) Θ(z,t)→0, z→−∞, t>0,

(7) Θ(z,0)=0,−∞<z≤d,

where [37,38]

(8) $$K_{1}(z)=K_{1,1}e^{\gamma^{*}z/d},\;\gamma^{*}=\ln(K_{1,2}K_{1,1}^{-1}),\;0\leq z\leq d,$$

and $K_{1,1}$, $K_{1,2}$ $K_{2}$ are the coefficients of thermal conductivity of FGM components and semi-space, respectively, $\rho_{l}$, $c_{l}$ are the density and specific heat of materials of the strip (l=1) and the semi-space (l=2), and $\gamma^{*}$ is the dimensionless gradient parameter.

Incorporating the following dimensionless parameters and variables:

(9) $$\zeta =\frac{z}{d},\;\tau =\frac{k_{1}t}{d^{2}},\;K^{*}=\frac{K_{2}}{K_{1,1}},\;k^{*}=\frac{k_{2}}{k_{1}},\;\Theta^{*}=\frac{\Theta }{\Theta_{0}},\;Bi=\frac{hd}{K_{1,1}},$$

where

(10) $$k_{1}=\frac{K_{1,1}}{c_{1}\rho_{1}},\;k_{2}=\frac{K_{2}}{c_{2}\rho_{2}},\;\Theta_{0}=\frac{q_{0}d}{K_{1,1}},$$

Equations (1)–(8) were written in the form:

(11) $$\frac{\partial^{2}\Theta^{*}(\zeta ,\tau )}{\partial \zeta^{2}}+\gamma^{*}\frac{\partial \Theta^{*}(\zeta ,\tau )}{\partial \zeta }-e^{-\gamma^{*}\zeta }\frac{\partial \Theta^{*}(\zeta ,\tau )}{\partial \tau }=0,\;0<\zeta <1,\;\tau >0,$$

(12) $$\frac{\partial^{2}\Theta^{*}(\zeta ,\tau )}{\partial \zeta^{2}}-\frac{1}{k_{2}^{*}}\frac{\partial \Theta^{*}(\zeta ,\tau )}{\partial \tau }=0,\;\zeta <0,\;\tau >0,$$

(13) $$\Theta^{*}(0^{+},\tau )=\Theta^{*}(0^{-},\tau ),\;\tau >0,$$

(14) $$K^{*}{\frac{\partial \Theta^{*}(\zeta ,\tau )}{\partial \zeta }|}_{\zeta =0^{-}}-{\frac{\partial \Theta^{*}(\zeta ,\tau )}{\partial \zeta }|}_{\zeta =0^{+}}=1,\;\tau >0,$$

(15) $${e^{\gamma^{*}}\frac{\partial \Theta^{*}(\zeta ,\tau )}{\partial \zeta }|}_{\zeta =1}+Bi\;\Theta^{*}(1,\tau )=0,\;\tau >0,$$

(16) $$\Theta^{*}(\zeta ,\tau )\to 0,\;\zeta \to -\infty ,\;\tau >0,$$

(17) $$\Theta^{*}(\zeta ,0)=0,\;\left|\zeta \right|<\infty .$$

## 3. Exact Solution

Using the Laplace integral transform [39]:

(18) Θ¯*(ζ,p)≡L[Θ*(ζ,τ);p]=∫0∞Θ*(ζ,τ)e−pτdτ, Rep≥0,

to the boundary-value problem (11)–(17), the following boundary problem was obtained with two ordinary differential equations:

(19) $$\frac{d^{2}{\bar{\Theta }}^{*}(\zeta ,p)}{d\zeta^{2}}+\gamma^{*}\frac{d{\bar{\Theta }}^{*}(\zeta ,p)}{d\zeta }-pe^{-\gamma^{*}\zeta }{\bar{\Theta }}^{*}(\zeta ,p)=0,\;0<\zeta <1,$$

(20) $$\frac{d^{2}{\bar{\Theta }}^{*}(\zeta ,p)}{d\zeta^{2}}-\frac{p}{k_{2}^{*}}{\bar{\Theta }}^{*}(\zeta ,p)=0,\;\zeta <0,$$

(21) $${\bar{\Theta }}^{*}(0^{+},p)={\bar{\Theta }}^{*}(0^{-},p),$$

(22) $${K^{*}\frac{d{\bar{\Theta }}^{*}(\zeta ,p)}{d\zeta }|}_{\zeta =0^{-}}-{\frac{d{\bar{\Theta }}^{*}(\zeta ,p)}{d\zeta }|}_{\zeta =0^{+}}=\frac{1}{p},$$

(23) $${e^{\gamma^{*}}\frac{d{\bar{\Theta }}^{*}(\zeta ,p)}{d\zeta }|}_{\zeta =1}+Bi\;{\bar{\Theta }}^{*}(1,p)=0,$$

(24) $${\bar{\Theta }}^{*}(\zeta ,p)\to 0,\;\zeta \to -\infty .$$

The solution to the boundary problem (19)–(24) has the form:

(25) $${\bar{\Theta }}^{*}(\zeta ,p)=\frac{e^{-\frac{1}{2}\gamma^{*}\zeta }\Delta_{1}(\zeta ,p)}{p\sqrt{p}\;\Delta (p)},\;0\leq \zeta \leq 1,\;{\bar{\Theta }}^{*}(\zeta ,p)=\frac{\Delta_{2}(\zeta ,p)}{p\sqrt{p}\;\Delta (p)},\;\zeta \leq 0,$$

where

(26) $$\Delta_{1}(\zeta ,p)=A_{1}(p{)I}_{1}\left(\frac{2}{\gamma^{*}}e^{-\frac{1}{2}\gamma^{*}\zeta }\sqrt{p}\right)+{Β}_{1}(p{)K}_{1}\left(\frac{2}{\gamma^{*}}e^{-\frac{1}{2}\gamma^{*}\zeta }\sqrt{p}\right),\;\Delta_{2}(\zeta ,p)=A_{2}(p)e^{\sqrt{\frac{p}{k^{*}}}\;\zeta },$$

(27) $$\Delta (p)=A_{1}(p)\left[I_{0}\left(\frac{2}{\gamma^{*}}\sqrt{p}\right)+\varepsilon \;I_{1}\left(\frac{2}{\gamma^{*}}\sqrt{p}\right)\right]-B_{1}(p)\left[K_{0}\left(\frac{2}{\gamma^{*}}\sqrt{p}\right)-\varepsilon \;K_{1}\left(\frac{2}{\gamma^{*}}\sqrt{p}\right)\right],$$

(28) $$A_{1}(p)=K_{0}\left(\frac{2}{\gamma^{*}}e^{-\frac{1}{2}\gamma^{*}}\sqrt{p}\right)+Bi\;\frac{e^{-\frac{1}{2}\gamma^{*}}}{\sqrt{p}}K_{1}\left(\frac{2}{\gamma^{*}}e^{-\frac{1}{2}\gamma^{*}}\sqrt{p}\right),$$

(29) $$B_{1}(p)=I_{0}\left(\frac{2}{\gamma^{*}}e^{-\frac{1}{2}\gamma^{*}}\sqrt{p}\right)-Bi\;\frac{e^{-\frac{1}{2}\gamma^{*}}}{\sqrt{p}}I_{1}\left(\frac{2}{\gamma^{*}}e^{-\frac{1}{2}\gamma^{*}}\sqrt{p}\right),$$

(30) $$A_{2}(p)=A_{1}(p{)I}_{1}\left(\frac{2}{\gamma^{*}}\sqrt{p}\right)+B_{1}(p{)K}_{1}\left(\frac{2}{\gamma^{*}}\sqrt{p}\right),$$

(31) $$\varepsilon =\frac{K^{*}}{\sqrt{k^{*}}},$$

where $I_{n}(x)$, $K_{n}(x)$ are modified Bessel’s functions of the *n*th (n=0,1) order of the first and second kind, respectively [40].

Applying the inverse Laplace transform [39]:

(32) Θ∗(ζ,τ)≡L−1[Θ¯∗(ζ,p); τ]=12πi∫ω−i ∞ω+i ∞Θ¯∗(ζ,p)epτdp, ω≡Rep>0, i≡−1,

to the transformed solution in Equations (25)–(31), and by carrying out the integration on the plane of the complex variable p, according to the methodology described in the articles [10,35], with account of the following relations [40]:

(33) $$I_{0}(\pm ix)=J_{0}(x),\;K_{0}(\pm ix)=-0.5\pi [Y_{0}(x)\pm iJ_{0}\left(x\right)],$$

(34) $$I_{1}(\pm ix)=\pm iJ_{1}(ix),\;K_{1}(\pm ix)=0.5\pi [J_{1}(x)\pm iY_{1}\left(x\right)],$$

where $J_{n}(x)$ and $Y_{n}(x)$ are the Bessel functions of the *n*th (n=0,1) order of the first and second kind, respectively, the dimensionless temperature rises in the strip and semi-space were obtained in the form:

(35) $$\Theta^{*}(\zeta ,\tau )=\vartheta (\zeta )-\frac{2}{\pi }\varepsilon \;e^{-\frac{1}{2}\gamma^{*}\zeta }\int_{0}^{\infty }F(x)G_{1}(\zeta ,x)e^{-x^{2}\tau }dx,\;0\leq \zeta \leq 1,\;\tau \geq 0,$$

(36) $$\Theta^{*}(\zeta ,\tau )=\vartheta_{0}-\frac{2}{\pi }\int_{0}^{\infty }F(x)G_{2}(\zeta ,x)e^{-x^{2}\tau }dx,\;\zeta \leq 0,\;\tau \geq 0,$$

where

(37) $$F(x)=\frac{\Psi (x)}{x^{2}\{{\left[\Phi (x)\right]}^{2}+{\left[\varepsilon \;\Psi (x)\right]}^{2}\}},$$

(38) $$G_{1}(\zeta ,x)=J(x)Y_{1}\left(\frac{2}{\gamma^{*}}e^{-\frac{1}{2}\gamma^{*}\zeta }x\right)-Y(x)J_{1}\left(\frac{2}{\gamma^{*}}e^{-\frac{1}{2}\gamma^{*}\zeta }x\right),$$

(39) $$G_{2}(\zeta ,x)=\varepsilon \;\Psi (x)\cos\left(\frac{\zeta }{\sqrt{k^{*}}}x\right)-\Phi (x)\sin\left(\frac{\zeta }{\sqrt{k^{*}}}x\right),$$

(40) $$\Phi (x)=J(x)Y_{0}\left(\frac{2}{\gamma^{*}}x\right)-Y(x)J_{0}\left(\frac{2}{\gamma^{*}}x\right),$$

(41) $$\Psi (x)=J(x)Y_{1}\left(\frac{2}{\gamma^{*}}x\right)-Y(x)J_{1}\left(\frac{2}{\gamma^{*}}x\right),$$

(42) $$J(x)=J_{0}\left(\frac{2}{\gamma^{*}}e^{-\frac{1}{2}\gamma^{*}}x\right)-Bi\;\frac{e^{-\frac{1}{2}\gamma^{*}}}{x}J_{1}\left(\frac{2}{\gamma^{*}}e^{-\frac{1}{2}\gamma^{*}}x\right),$$

(43) $$Y(x)=Y_{0}\left(\frac{2}{\gamma^{*}}e^{-\frac{1}{2}\gamma^{*}}x\right)-Bi\;\frac{e^{-\frac{1}{2}\gamma^{*}}}{x}Y_{1}\left(\frac{2}{\gamma^{*}}e^{-\frac{1}{2}\gamma^{*}}x\right),$$

(44) $$\vartheta (\zeta )={\left(\gamma^{*}Bi\right)}^{-1}[\gamma^{*}-Bi(e^{-\gamma^{*}}-e^{-\gamma^{*}\zeta })],\;\vartheta_{0}\equiv \vartheta (0)={\left(\gamma^{*}Bi\right)}^{-1}[\gamma^{*}-Bi(e^{-\gamma^{*}}-1)].$$

On the contact surface ζ=0, from Equations (38) and (39) follows that $G_{1}(0,x)=\Psi (x)$, $G_{2}(0,x)=\varepsilon \;\Psi (x)$. Then, the solutions (35) and (36) have the form:

(45) $$\Theta^{*}(0^{+},\tau )=\Theta^{*}(0^{-},\tau )\equiv \Theta^{*}(\tau )=\vartheta_{0}-\frac{2}{\pi }\varepsilon \int_{0}^{\infty }F(x)\Psi (x)e^{-x^{2}\tau }dx,\;\tau \geq 0,$$

which confirms the fulfillment of the boundary condition (13). Differentiating solutions (35)–(44) with respect to the variable ζ, with account of the derivatives [40]:

(46) $$J_{1}^{\prime }(x)=J_{0}(x)-x^{-1}J_{1}(x),\;Y_{1}^{\prime }(x)=Y_{0}(x)-x^{-1}Y_{1}(x),$$

the dimensionless intensities of heat fluxes absorbed by the strip and the semi-space were found:

(47) $$\frac{\partial \Theta^{*}(\zeta ,\tau )}{\partial \zeta }=-e^{-\gamma^{*}\zeta }+\frac{2}{\pi }\varepsilon \;e^{-\gamma^{*}\zeta }\int_{0}^{\infty }xF(x){\hat{G}}_{1}(\zeta ,x)e^{-x^{2}\tau }dx,\;0\leq \zeta \leq 1,\;\tau \geq 0,$$

(48) $$K^{*}\frac{\partial \Theta^{*}(\zeta ,\tau )}{\partial \zeta }=\frac{2}{\pi }\varepsilon \int_{0}^{\infty }xF(x){\hat{G}}_{2}(\zeta ,x)e^{-x^{2}\tau }dx,\;\zeta \leq 0,\;\tau \geq 0,$$

where

(49) $${\hat{G}}_{1}(\zeta ,x)=J(x)Y_{0}\left(\frac{2}{\gamma^{*}}e^{-\frac{1}{2}\gamma^{*}\zeta }x\right)-Y(x)J_{0}\left(\frac{2}{\gamma^{*}}e^{-\frac{1}{2}\gamma^{*}\zeta }x\right),$$

(50) $${\hat{G}}_{2}(\zeta ,x)=\varepsilon \;\Psi (x)\sin\left(\frac{\zeta }{\sqrt{k^{*}}}x\right)+\Phi (x)\cos\left(\frac{\zeta }{\sqrt{k^{*}}}x\right),$$

and $K^{*}$, $k^{*}$ are the dimensionless thermal conductivity and diffusivity of the system, respectively, as determined by Equation (9).

Substituting ζ=0 in Equations (49) and (50), we found ${\hat{G}}_{1}(0,x)={\hat{G}}_{2}(0,x)=\Phi (x)$ and Formulas (47) and (48) take the form:

(51) $${\frac{\partial \Theta^{*}(\zeta ,\tau )}{\partial \zeta }|}_{\zeta =0^{+}}\equiv q_{1}^{*}(\tau )=-1+\frac{2}{\pi }\varepsilon \int_{0}^{\infty }xF(x)\Phi (x)e^{-x^{2}\tau }dx,\;\tau \geq 0,$$

(52) $${K^{*}\frac{\partial \Theta^{*}(\zeta ,\tau )}{\partial \zeta }|}_{\zeta =0^{-}}\equiv q_{2}^{*}(\tau )=\frac{2}{\pi }\varepsilon \int_{0}^{\infty }xF(x)\Phi (x)e^{-x^{2}\tau }dx,\;\tau \geq 0.$$

From Equations (51) and (52), it is easy to obtain the confirmation of the fulfillment of the boundary condition (14). For the purpose of checking the boundary condition (15) on the free surface of the strip ζ=1, from relations (35), (38), (44), (47) and (49), it was found:

(53) $$\Theta^{*}(1,\tau )=\frac{1}{Bi}-\frac{2}{\pi }\varepsilon \;e^{-\frac{1}{2}\gamma^{*}}\int_{0}^{\infty }F(x)G_{1}(1,x)e^{-x^{2}\tau }dx,\;\tau \geq 0,$$

(54) $${\frac{\partial \Theta^{*}(\zeta ,\tau )}{\partial \zeta }|}_{\zeta =1}=-e^{-\gamma^{*}}+\frac{2}{\pi }\varepsilon \;e^{-\gamma^{*}}\int_{0}^{\infty }xF(x){\hat{G}}_{1}(1,x)e^{-x^{2}\tau }dx,\;\tau \geq 0,$$

where

(55) $$G_{1}(1,x)=J(x)Y_{1}\left(\frac{2}{\gamma^{*}}e^{-\frac{1}{2}\gamma^{*}}x\right)-Y(x)J_{1}\left(\frac{2}{\gamma^{*}}e^{-\frac{1}{2}\gamma^{*}}x\right),$$

(56) $${\hat{G}}_{1}(1,x)=J(x)Y_{0}\left(\frac{2}{\gamma^{*}}e^{-\frac{1}{2}\gamma^{*}}x\right)-Y(x)J_{0}\left(\frac{2}{\gamma^{*}}e^{-\frac{1}{2}\gamma^{*}}x\right).$$

Bearing in mind Equations (53)–(56) and the left-hand side of the boundary condition (15), it was written in the form:

(57) $${e^{\gamma^{*}}\frac{\partial \Theta^{*}(\zeta ,\tau )}{\partial \zeta }|}_{\zeta =1}+Bi\;\Theta^{*}(1,\tau )=\frac{2}{\pi }\varepsilon \;e^{-\gamma^{*}}\int_{0}^{\infty }F(x)[x{\hat{G}}_{1}(1,x)-e^{-\frac{1}{2}\gamma^{*}}Bi\;G_{1}(1,x)]e^{-x^{2}\tau }dx,\;\;\tau >0.$$

Substituting the functions J(x) (42) and Y(x) (43) to Equations (55) and (56), the relations were determined [40]:

(58) $$G_{1}(1,x)=J_{0}\left(\frac{2}{\gamma^{*}}e^{-\frac{1}{2}\gamma^{*}}x\right)Y_{1}\left(\frac{2}{\gamma^{*}}e^{-\frac{1}{2}\gamma^{*}}x\right)-Y_{0}\left(\frac{2}{\gamma^{*}}e^{-\frac{1}{2}\gamma^{*}}x\right)J_{1}\left(\frac{2}{\gamma^{*}}e^{-\frac{1}{2}\gamma^{*}}x\right)\equiv -\frac{\gamma^{*}e^{\frac{1}{2}\gamma^{*}}}{\pi \;x},$$

(59) $${\hat{G}}_{1}(1,x)=e^{-\frac{1}{2}\gamma^{*}}Bi\;x^{-1}G_{1}(1,x).$$

Proving the zero value of the integral in the right side of Equation (57), and thus, the boundary condition (15) is met.

The fulfillment of the boundary condition (16) and the initial condition (17) were verified numerically.

## 4. Some Special Cases of Solution

Usage of the exact solutions (35) and (36) requires the numerical integration over a semi-limited interval, which requires the application of appropriate, sometimes complex, software. In this section, the asymptotic solutions (for small and large values of the Fourier number τ) will be developed, which are devoid of this problem. In addition, the exact solutions for the temperature generated in the strip and semi-space in the process of frictional heating during braking with constant deceleration will be presented.

*Small values of Fourier number* τ (*large values of the Laplace transform parameter* p). Including Formulas (26)–(30), the asymptotes of the modified Bessel functions for large values of arguments [40]:

(60) $$I_{n}(x)≅\frac{e^{x}}{\sqrt{2\pi x}},\;K_{n}(x)≅\sqrt{\frac{\pi }{2x}}\;e^{-x},\;n=0,\;1,$$

where the transformed solutions (25) were written in the form:

(61) $${\bar{\Theta }}^{*}(\zeta ,p)≅\frac{e^{-\frac{1}{4}\gamma^{*}\zeta }}{\left(1+\varepsilon \right)}\frac{e^{-\alpha \sqrt{p}}}{p\sqrt{p}},\;\;0\leq \zeta <1,\;{\bar{\Theta }}^{*}(\zeta ,p)≅\frac{1}{\left(1+\varepsilon \right)}\frac{e^{-\zeta^{*}\sqrt{p}}}{p\sqrt{p}},\;\zeta \leq 0,$$

where

(62) $$\alpha =\frac{2}{\gamma^{*}}(1-e^{-\frac{1}{2}\gamma^{*}\zeta }),\;\zeta^{*}=\frac{\left|\zeta \right|}{\sqrt{k^{*}}}.$$

Proceeding in the transforms (61) to the originals by means of the relation [41]:

(63) $$L^{-1}\left[\frac{e^{-\alpha \sqrt{p}}}{p\sqrt{p}};\tau \right]=2\sqrt{\tau \;}\mathrm{ierfc}\left(\frac{\alpha }{2\sqrt{\tau }}\right),\;\alpha \geq 0,$$

where $\mathrm{ierfc}(x)=\pi^{-0.5}e^{-x^{2}}-x\;\mathrm{erfc}(x)$, erfc(x)=1−erf(x), and erf(x) is the Gauss error function [40]. Asymptotes of the dimensionless temperature rise at the initial moments of the heating process were found:

(64) $$\Theta^{*}(\zeta ,\tau )≅\frac{2\sqrt{\tau \;}e^{-\frac{1}{4}\gamma^{*}\zeta }}{\left(1+\varepsilon \right)}\mathrm{ierfc}\left(\frac{\alpha }{2\sqrt{\tau }}\right),\;0\leq \zeta \leq 1,\;0\leq \tau <<1,$$

(65) $$\Theta^{*}(\zeta ,\tau )≅\frac{2\sqrt{\tau }}{(1+\varepsilon )}\mathrm{ierfc}\left(\frac{\zeta^{*}}{2\sqrt{\tau }}\right),\;\zeta \leq 0,\;0\leq \tau <<1.$$

On the contact surface ζ=0, with consideration of the parameter α definition in Equation (62), from Equations (64) and (65), the following was obtained:

(66) $$\Theta^{*}(0^{+},\tau )=\Theta^{*}(0^{-},\tau )=\Theta^{*}(\tau )≅\frac{2}{\left(1+\varepsilon \right)}\sqrt{\frac{\tau }{\pi }}.$$

In the case of homogeneous strip ($\gamma^{*}\to 0$) from Equation (62), we determined α→ζ and the solutions (64) and (65) take the form of a known solution of the problem for two homogeneous semi-spaces under uniform sliding [5]:

(67) $$\Theta^{*}(\zeta ,\tau )=\frac{2\sqrt{\tau \;}}{\left(1+\varepsilon \right)}\mathrm{ierfc}\left(\frac{\zeta }{2\sqrt{\tau }}\right),\;\zeta \geq 0,\;\tau \geq 0,$$

(68) $$\Theta^{*}(\zeta ,\tau )=\frac{2\sqrt{\tau }}{(1+\varepsilon )}\mathrm{ierfc}\left(-\frac{\zeta }{2\sqrt{k^{*}\tau }}\right),\;\zeta \geq 0,\;\tau \geq 0.$$

For ζ=0 from Equations (67) and (68), we received also the solution (66) to determine the temperature on the contact surface.

Analyzing the obtained asymptotic solutions (64) and (65), it can be noticed that at the beginning of the friction heating process, the effect of convective cooling on the free surface of the strip on the temperature of both elements is insignificant, and the gradient nature of the material affects the temperature only inside the layer.

*Large values of Fourier number* τ (*small values of Laplace parameter* p). For small values of arguments, the modified Bessel function behaves as follows [40]:

(69) $$I_{0}(x)≅1,\;I_{1}(x)≅0.5x,\;K_{0}(x)≅-\ln(x),\;K_{1}(x)≅x^{-1}.$$

Considering the asymptotes (69) in Equations (26)–(30), the transform solutions (26) were presented in the form:

(70) $${\bar{\Theta }}^{*}(\zeta ,p)≅\frac{\phi^{*}(\zeta )}{p(\sqrt{p}+a)},\;\;0\leq \zeta \leq 1,\;{\bar{\Theta }}^{*}(\zeta ,p)≅\frac{e^{-\zeta^{*}\sqrt{p}}}{\varepsilon p(\sqrt{p}+a)},\;\;\zeta \leq 0,$$

where

(71) $$\phi^{*}(\zeta )=\frac{\phi (\zeta )}{\varepsilon \phi_{0}},\;a=\frac{\gamma^{*}Bi}{\varepsilon \phi_{0}},$$

(72) $$\phi (\zeta )=\gamma^{*}+Bi(e^{-\gamma^{*}\zeta }-e^{-\gamma^{*}}),\;\phi_{0}\equiv \phi (0)=\gamma^{*}+Bi(1-e^{-\gamma^{*}}).$$

Taking into consideration the relation [41]:

(73) $$L^{-1}\left[\frac{a\;e^{-b\sqrt{p}}}{p(\sqrt{p}+a)};\tau \right]=\mathrm{erfc}\left(\frac{b}{2\sqrt{\tau }}\right)-e^{ab+a^{2}\tau }\mathrm{erfc}\left(\frac{b}{2\sqrt{\tau }}+a\sqrt{\tau }\right),\;a>0,\;b\geq 0,$$

the following asymptotes of dimensionless temperature rise for large values of the Fourier number, and τ were found:

(74) $$\Theta^{*}(\zeta ,\tau )≅a^{-1}\phi^{*}(\zeta )[1-e^{a^{2}\tau }\mathrm{erfc}(a\sqrt{\tau })],\;0\leq \zeta \leq 1,\;\tau >>1,$$

(75) $$\Theta^{*}(\zeta ,\tau )≅\frac{1}{a\varepsilon }\left[\mathrm{erfc}\left(\frac{\zeta^{*}}{2\sqrt{\tau }}\right)-e^{a\zeta^{*}+a^{2}\tau }\mathrm{erfc}\left(\frac{\zeta^{*}}{2\sqrt{\tau }}+a\sqrt{\tau }\right)\right],\;\zeta \leq 0,\;\tau >>1.$$

On the friction surface ζ=0 from Equations (62) and (72) follows that $\zeta^{*}=0$ and $\phi^{*}(0)=\varepsilon^{-1}$, and from the solutions (74) and (75), yields:

(76) $$\Theta^{*}(0^{+},\tau )=\Theta^{*}(0^{-},\tau )\equiv \Theta^{*}(\tau )≅\;(a\varepsilon {)}^{-1}[1-e^{a^{2}\tau }\mathrm{erfc}(a\sqrt{\tau })],\;\tau >>1,$$

where parameters ε and a were designated on the basis of Equations (31) and (71).

Bearing in mind the values of limits:

(77) $$\underset{\gamma^{*}\to 0}{\lim}a=\frac{Bi}{\varepsilon }\underset{\gamma^{*}\to 0}{\lim}\frac{\gamma^{*}}{\left[\gamma^{*}+Bi(1-e^{-\gamma^{*}})\right]}=\frac{Bi}{\varepsilon (1+Bi)},$$

(78) $$\underset{\gamma^{*}\to 0}{\lim}a^{-1}\beta (\zeta )=\frac{1}{Bi}\underset{\gamma^{*}\to 0}{\lim}\frac{\left[\gamma^{*}+Bi(e^{-\gamma^{*}\zeta }-e^{-\gamma^{*}})\right]}{\gamma^{*}}=\frac{1+Bi(1-\zeta )}{Bi},$$

from the Equations (74) and (75), the solution were obtained to determine the temperature in the considered system with a homogeneous strip [10].

*Linearly decreasing the time profile of specific friction power.* Demonstrated above, the exact solutions (35)–(44) were found for the invariable specific friction power over time, $q(t)=q_{0}$, t≥0 in the boundary condition (4). In the relevant thermal problems of friction concerning the modeling of the frictional heating process during braking with a constant deceleration, the temporal profile of specific friction power has the form [1]:

(79) $$\hat{q}(t)={\hat{q}}_{0}q^{*}(t),\;{\hat{q}}_{0}=2q_{0},\;q^{*}(t)=1-t\;t_{s}^{-1},\;0\leq t\leq t_{s},$$

where $t_{s}$ is the stop moment of the vehicle. Dimensionless temperature rise ${\hat{\Theta }}^{*}(\zeta ,\tau )$, corresponding to the specific power of friction (79), was found based on the Duhamel’s theorem [42]:

(80) $${\hat{\Theta }}^{*}(\zeta ,\tau )=\frac{\partial }{\partial \tau }\int_{0}^{\tau }q^{*}(\tau -s)\Theta^{*}(\zeta ,s)ds,\;\zeta \geq 0,\;0\leq \tau \leq \tau_{s},$$

where $\Theta^{*}(\zeta ,\tau )$ is the dimensionless temperature rise (35)–(44) and function $q^{*}(\tau )$:

(81) $$q^{*}(\tau )=1-\tau \;\tau_{s}^{-1},\;0\leq \tau \leq \tau_{s},\;\tau_{s}=k_{1}t_{s}d^{-2}.$$

Substituting solutions (35) and (36) and function $q^{*}(\tau )$ (81) to the right side of Equation (80) yields:

(82) $${\hat{\Theta }}^{*}(\zeta ,\tau )=\vartheta (\zeta )q^{*}(\tau )-\frac{2}{\pi }\varepsilon \;e^{-\frac{1}{2}\gamma^{*}\zeta }\int_{0}^{\infty }F(x)G_{1}(\zeta ,x)P(\tau ,x)dx,\;0\leq \zeta \leq 1,\;0\leq \tau \leq \tau_{s},$$

(83) $${\hat{\Theta }}^{*}(\zeta ,\tau )=\vartheta_{0}q^{*}(\tau )-\frac{2}{\pi }\int_{0}^{\infty }F(x)G_{2}(\zeta ,x)P(\tau ,x)dx,\;\zeta \leq 0,\;0\leq \tau \leq \tau_{s},$$

where

(84) $$P(\tau ,x)=e^{-x^{2}\tau }-\frac{1}{x^{2}\tau_{s}}(1-e^{-x^{2}\tau }),$$

and the constant $\vartheta_{0}$ and functions ϑ(ζ), F(x), $G_{l}(\zeta ,x)$, l=1,2 were determined from Equations (37)–(44).

## 5. Numerical Analysis

Approval of the developed calculation model was performed for a friction system, in which the strip is made of a two-component FGM, and the counterbody is homogeneous (cast iron ChNMKh). The base of the FGM is zirconium dioxide ZrO_2_, and on the second component of material the titanium alloy Ti-6Al-4V was selected. Properties of these materials at the initial temperature $T_{0}=20\;{}^{\circ}C$ are given in Table 1. For the same volume fraction of ZrO_2_ and Ti-6Al-4V, effective specific heat and density of the strip material amounted to $c_{1}=495.55\;\;J\;{\mathrm{kg}}^{-1}K^{-1}$ and $\rho_{1}=\;5266.98\;\mathrm{kg}\;m^{-3}$, respectively. By means of Equation (8), the dimensionless gradient of selected FGM was also established $\gamma^{*}=1.26$. The rest dimensionless input parameters for the calculations are spatial variable ζ, Fourier number τ and Biot number Bi (9).

**Figure 2:**
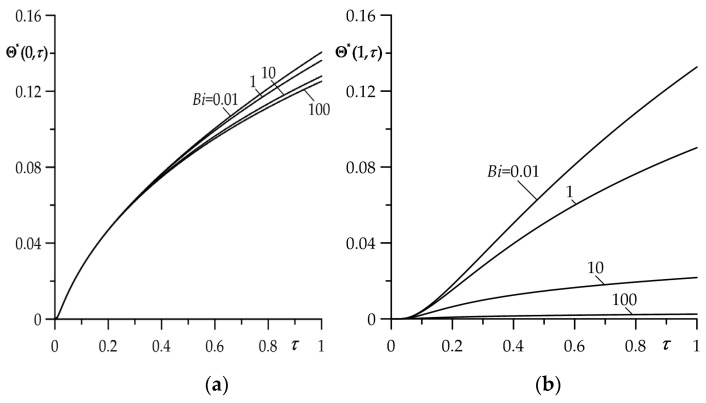
Evolutions of dimensionless temperature rise $\Theta^{*}$ for selected values of Biot number: (**a**) on the contact surface ζ=0; (**b**) on the free surface of the strip ζ=1.

**Figure 3:**
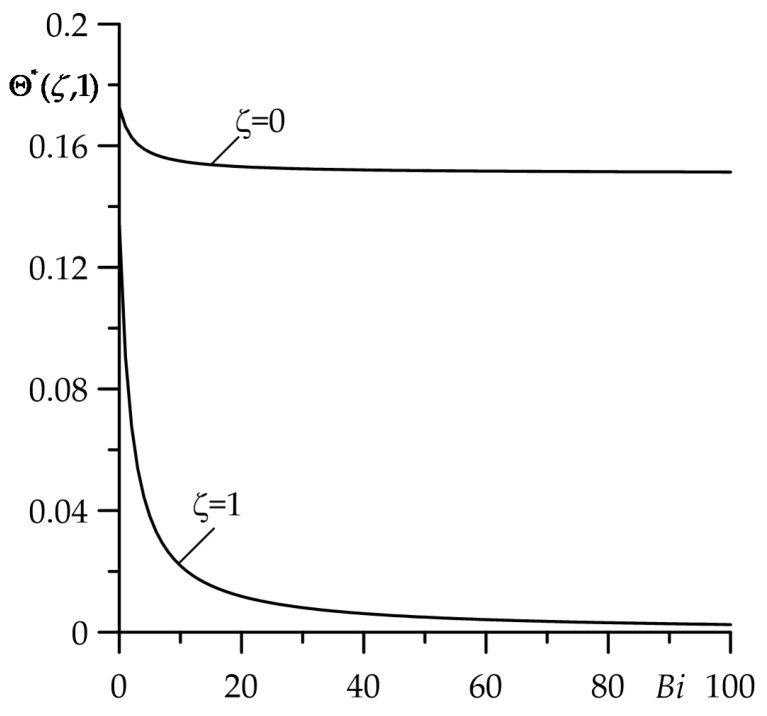
Dependence of dimensionless temperature rise $\Theta^{*}$ on the Biot number Bi on the surfaces ζ=0 and ζ=1 for τ=1.

**Figure 4:**
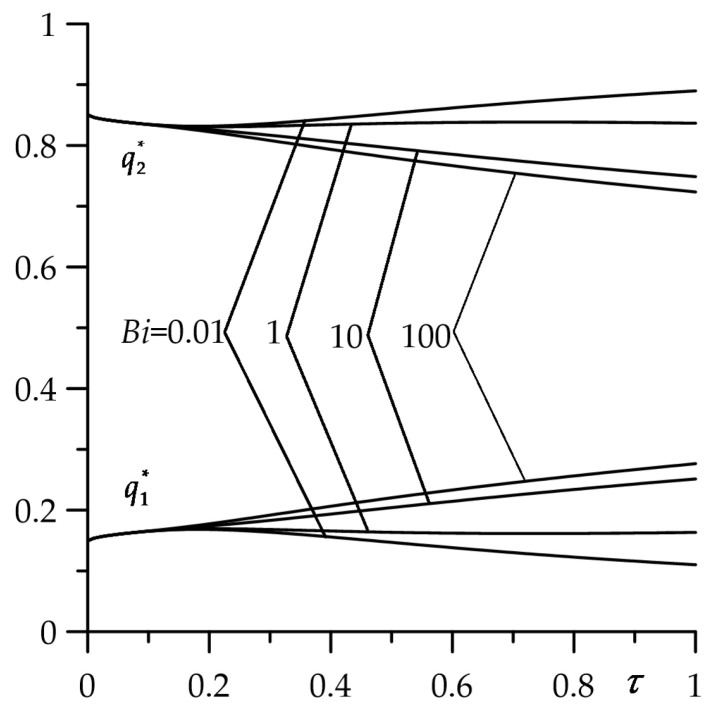
Evolutions of dimensionless intensities of a $q_{l}^{*}(\tau )$ heat fluxes absorbed by FGM strip (l=1) and homogeneous half-space (l=2) for selected values of the Biot number Bi.

**Figure 5:**
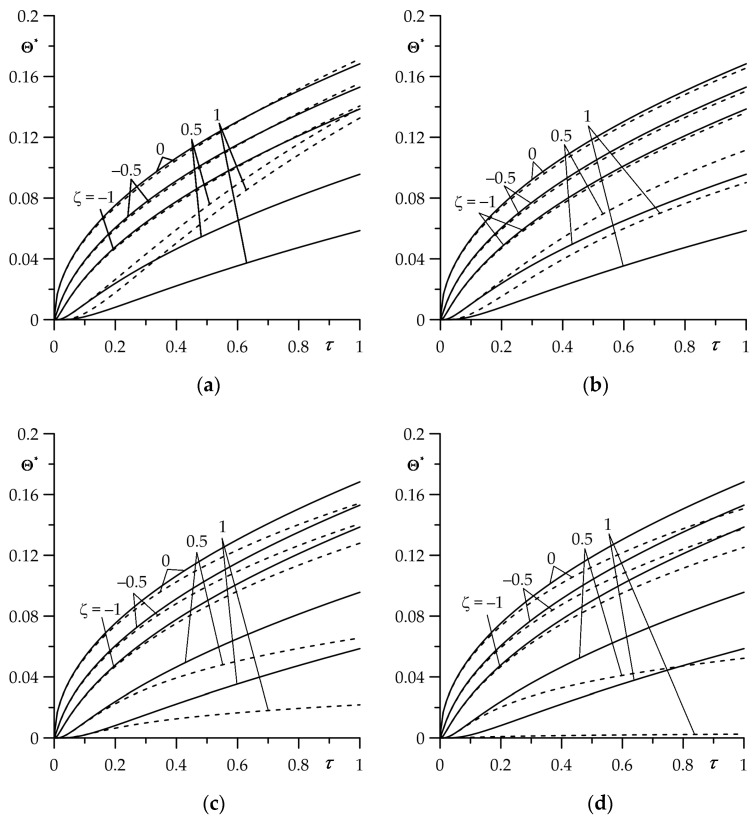
Evolutions of dimensionless temperature rise $\Theta^{*}$ for different values of dimensionless spatial variable ζ for (**a**) Bi=0.01; (**b**) Bi=1; (**c**) Bi=10; and (**d**) Bi=100. The solid curves are the exact solution (35)–(44), and the dashed curves are the asymptotic solution (64) and (65) for small values of the Fourier number τ.

**Figure 6:**
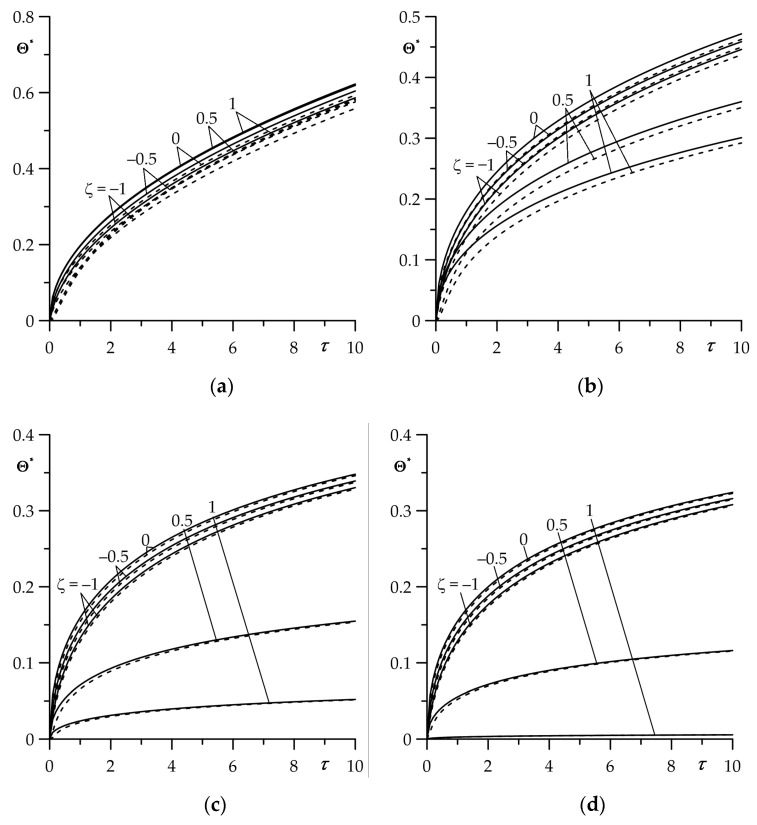
Evolutions of dimensionless temperature rise $\Theta^{*}$ for different values of dimensionless spatial variable ζ for (**a**) Bi=0.01; (**b**) Bi=1; (**c**) Bi=10; and (**d**) Bi=100. The solid curves are the exact solution(35)–(44), and the dashed curves are the asymptotic solution (74) and (75) for large values of the Fourier number τ.

The aim of this numerical analysis was to establish the qualitative effect of the intensity of the convective heat exchange with the environment (parameter Bi) on the free surface of the FGM strip z=d (ζ=1) on the temperature of friction system. It should be noted that the case Bi→0 corresponds to the thermal isolation of the surface ζ=1; however, for Bi→∞ on this surface during the whole process of heating, the initial temperature is sustained $T(d,t)=T_{0}$ ($\Theta^{*}(1,\tau )=0$). Results for dimensionless temperature rise $\Theta^{*}(\zeta ,\tau )$ (35)–(44) and the intensity of the heat fluxes $q_{l}^{*}(\tau )$, l=1,2 (51) and (52) obtained by means of the numerical integral procedure QAGI from the package QUADPACK [43] were presented in Figure 2, Figure 3, Figure 4, Figure 5 and Figure 6.

Influence of the Biot number Bi value on the evolution of $\Theta^{*}(\zeta ,\tau )$ is shown in Figure 2. A slight decrease in temperature on the friction surface ζ=0 with the increase in Bi becomes visible for τ≥0.6 (Figure 2a). While the free surface ζ=1 of the FGM strip is more sensitive to changes in the Biot number. Growth of the convective cooling intensity causes a significant decrease in the temperature of this surface much earlier, at τ≥0.1 (Figure 2b). For the fixed value of Fourier number τ, the temperature on both surfaces drops for higher values of parameter Bi. This effect is most noticeable on the free surface of the FGM strip ζ=1.

The drop in temperature on the contact surface ζ=0 and the free surface ζ=1 of the strip with the increase in the Biot number Bi for the fixed value τ=1 is demontrated in Figure 3. The highest temperature on both surfaces is achieved for the assumption of the adiabatic (Bi=0) free strip surface. Next, growth of Bi causes cooling of the considered surfaces. An explicit decrease in temperature on the contact surface ζ=0 is visible for 0≤Bi≤10. A further increase in Bi shows practically no effect on the temperature on this surface.

Thus, when estimating the maximum (achievable at the contact surface ζ=0) temperature of the selected friction system, the cooling of the free strip surface should be taken into consideration for the values of the Biot number outside the specified range. If Bi>10, then the boundary condition (5) in the formulation to the thermal problem of friction has to be replaced by its simplified variant $T(d,t)=T_{0}$, t>0 ($\Theta^{*}(1,\tau )=0$ in the condition (15)). A decrease in Bi causes the drop in temperature on the surface ζ=1 to the level of the initial temperature $\Theta^{*}=0$, the most noticeable in the range 0≤Bi≤60.

Most of the heat generated by friction on the contact surface is absorbed by the semi-space (Figure 4). This is due to the much better thermal conductivity of cast iron compared to zirconium dioxide (Table 1). In the initial heating period (0<τ≤0.2), the strip absorbs ≈15%, and the semi-space the remaining ≈85% of the heat. The proportion of heat distribution between the strip and the half-space changes with the sliding time depending on the value of Bi. For small values of Biot number, the amount of heat absorbed by the strip decreases slightly in time of heating, achieving for τ=1 the values ≈10% and ≈13% for Bi=0.01 and Bi=1, respectively. At the same time, the amount of heat directed to the half-space increases proportionally. The growth of the cooling intensity on the free strip surface increases the heat absorbed by it for τ=1 to 20% and 27% for Bi=10 and Bi=100, respectively.

The comparison of temperature values found by means of exact solution (35)–(44) (solid lines) and asymptotic solutions (64) and (65) for small values of the Fourier number τ (dashed lines) are illustrated in Figure 5. Good agreement of the results for 0≤τ≤1, determined based on the exact and asymptotic solutions, take place on the contact surface ζ=0 and inside the semi-space for ζ=−0.5 and ζ=−1, for all four selected values of Bi. Whereas inside the FGM strip, using the asymptotic solution should be restricted to the range 0≤τ≤0.2.

Asymptotic solutions (74) and (75) for large values of τ allow for estimation of the temperature in the range 0≤τ≤10 both in the strip and in the half-space (Figure 6). The accuracy of such an estimation rises with the increasing intensity of convective cooling on the free strip surface. Such a good agreement of the temperature time profiles, found with the use of exact and asymptotic solutions, allows for extensive use of the latter in engineering calculations of the temperature mode for the selected friction pair. The advantage of the asymptotic solution is the lack of numerical integration, which occurs when using the exact solution (35)–(44).

**Figure 7:**
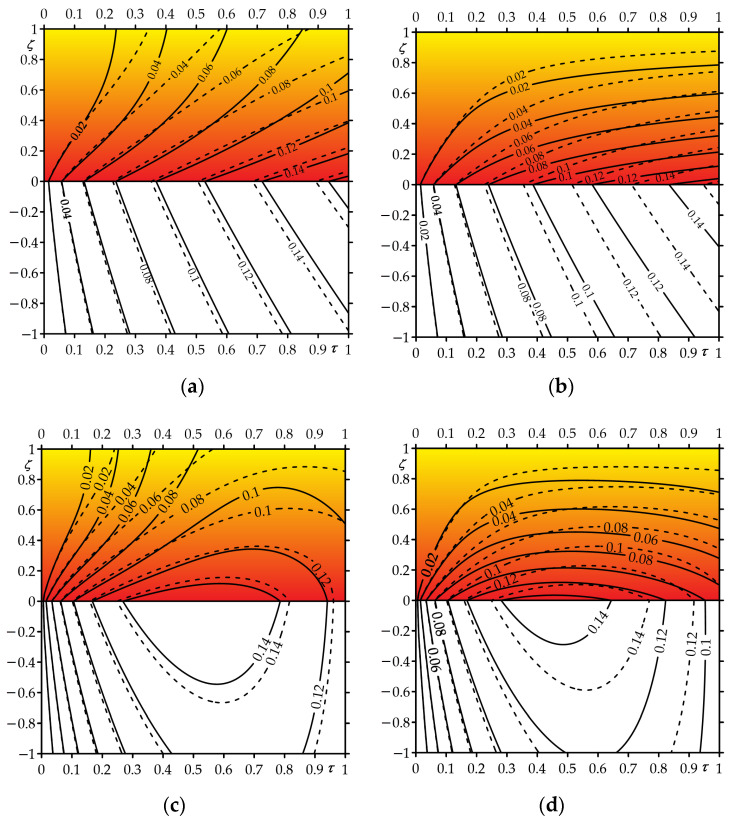
Isotherms of dimensionless temperature rise $\Theta^{*}(\zeta ,\tau )$ during sliding with specific friction surface: (**a**,**b**)—constant; (**c**,**d**)—linearly increasing in time for (**a**,**c**)—Bi=1; (**b**,**d**)—Bi=100. Solid lines—strip made of FGM ZrO_2_—Ti-6Al-4V, dashed lines—homogeneous strip made of ZrO_2_.

An influence of functionally graded material of the strip on the temperature field of the friction system is presented in Figure 7. Calculations were carried out based on the exact solutions for sliding under the constant (35)–(44) or linearly decreasing (82)–(84) temporal profile of specific friction power. Heating of the strip over the entire thickness is visible with low (Bi=1) intensity of convective cooling on the free strip surface (Figure 7a,c). However, when the Biot number is increased to the value Bi=100, the initial temperature is maintained on the free surface of the strip (Figure 7b,d).

Hence, we conclude that when determining such an important parameter as the effective depth of heat transfer during calculations of the temperature mode of the friction node [7], in the case Bi=1, the entire thickness of the strip should be taken, and in the case Bi=100, only that part of it should be taken that is determined by the distance from the contact surface at which the temperature is 5% of the maximum value.

The reduction in the temperature of the friction system as a result of the use of FGM is most noticeable near the contact surface during braking with a constant deceleration (Figure 7c,d). During uniform sliding with constant velocity, the temperature of the strip and the half-space at a fixed distance from the contact surface increase monotonically over the heating time (Figure 7a,b). By contrast, in the case of sliding with a linearly decreasing velocity, the temperature first quickly increases to the maximum value, after which the stage of slight cooling begins and lasts until the standstill (Figure 7c,d).

## 6. Conclusions

An analytical model was developed to simulate the processes of frictional heating on the contact surface and convection cooling on the free surface of the friction pair, in which one element was made of FGM and the other of a homogeneous material. Ignoring changes in the temperature gradient in directions parallel to the contact surface, the transient, one-dimensional temperature field in such a system was found from the exact solution of the thermal friction problem for the strip/half-space scheme at constant specific friction power. It was assumed that the two-component FGM strip has a thermal conductivity coefficient increasing exponentially along the thickness, and the material of the half-space is homogeneous. The friction thermal contact of the strip and the semi-space is perfect, and on the free surface of strip, the heat exchange with the surrounding environment takes place according to Newton’s law. In addition, the exact, asymptotic solutions were also obtained for small and large values of the Fourier number. Using the Duhamel’s formula and the solution at a constant specific friction power, appropriate solutions were determined with a linearly decreasing time profile of the specific friction power. This made it possible to simulate the frictional heating process during braking with a constant deceleration.

Numerical analysis was performed for a functionally graded strip (ZrO_2_—Ti-6Al-4V), sliding against the cast iron half-space (ChNMKh). The following was established:

(1) Applying of FGM for one element of the friction couple (strip) allows for a decrease in the temperature on the contact surface in comparison to the case of the homogeneous strip (zirconium dioxide);
(2) A convective heat exchange with the environment on the free surface of the strip causes a decrease in the temperature on the contact surface at the values of the Biot number 0≤Bi≤10. However, the greatest drop in temperature on the free surface of the strip occurs in the range of changes 0≤Bi≤60;
(3) Most part of the frictional heat is absorbed by the cast iron semi-space (≈85%) in the initial stage of the heating process. With the elapse of the slipping time and the increase in the cooling intensity of the free surface of strip, the amount of heat absorbed by the half-space decreases to 73% for τ=1 and Bi=100. The amount of heat directed to the FGM strip increases accordingly;
(4) Obtained asymptotic solutions for small and large values of the Fourier number τ can be used to quickly estimate the temperature of both elements of the system, with high accuracy. At the same time, the solution for large values of the Fourier number is useful for determining the temperature at any time during the friction heating process at τ>0;
(5) Convective cooling of the FGM strip allows for a reduction in the effective depth of heating, i.e., the distance from the contact surface at which the temperature of each element reaches significant values;
(6) The space–time distribution of isotherms in the strip and semi-space depends on the time profile of the specific friction power. With a constant friction power during sliding, the temperature monotonically increases with the increasing heating time (Fourier number τ). However, in the case of braking with constant deceleration, the temperature of the friction surface reaches its maximum value around half the stopping time $\tau_{s}$.

Summing up, the analysis carried out on the basis of the developed mathematical model of the frictional heating process showed that both the use of FGM and convection cooling in the process allow for an effective reduction in the temperature of the friction pair elements.

We would like to note that analytical solutions of one-dimensional friction thermal problems allow for estimating with good accuracy the maximum temperature of friction systems. This has been confirmed in many papers containing relevant experimental measurements [44,45]. These solutions are used to estimate the temperature on the nominal contact surface of the braking system (an average during one cycle of heating and cooling during disc rotation). It is one of the components to determine the maximum temperature in such a system, and the second component is the flash temperature. The maximum temperature is a design parameter for the development of a methodology for the initial selection of friction materials in various types of brake systems, including disc brake systems.

Such models regarding FGMs, proposed by us so far, assume the perfect thermal friction contact between the elements. This is fully justified when the friction surfaces of these elements are sufficiently smooth. However, in reality, these surfaces are rough, depending on the level of treatment and operating conditions. This causes the thermal resistance of the contact surface, and as a result, the appearance of a temperature jump on the friction surfaces. One of the approaches to solve this problem is to introduce into the formulation of the relevant problems, the conditions of imperfect thermal contact of friction. We plan to implement it at the next stage of our research, obtaining a solution to this problem in the case of friction pair elements made of FGM and investigating the effect of thermal resistance on temperature.

## Figures and Tables

**Table 1:** Materials’ properties [36].

Material	Thermal Conductivity ${\mathrm{Wm}}^{-1}K^{-1}$	Specific Heat $J\;{\mathrm{kg}}^{-1}K^{-1}$	Density $\;\mathrm{kg}\;m^{-3}$
ZrO_2_	$K_{1,1}=1.94$	$c_{1,1}=\;452.83$	$\rho_{1,1}=\;6102.16$
Ti-6Al-4V	$K_{1,2}=6.87$	$c_{1,2}=\;538.08$	$\rho_{1,2}=\;4431.79$
ChNMKh	$K_{2}=52.17$	$c_{2}=\;444.6$	$\rho_{2}=\;7100$

## Data Availability

No new data were created or analyzed in this study. Data sharing is not applicable to this article.

## Nomenclature

Bi	Biot number
c	Specific heat ($J\;{\mathrm{kg}}^{-1}K^{-1}$)
d	Thickness of the strip (m)
f	Coefficient of friction
h	Coefficient of convective heat exchange ($W\;m^{-2}K^{-1}$)
$I_{n}(\cdot )$	Modified Bessel functions of the *n*th order of the first kind
$J_{n}(\cdot )$	Bessel functions of the *n*th order of the first kind
$K_{n}(\cdot )$	Modified Bessel functions of the *n*th order of the second kind
k	Thermal diffusivity ($m^{2}s^{-1}$)
K	Thermal conductivity ($W\;m^{-1}K^{-1}$)
p	Parameter of the Laplace integral transform
$p_{0}$	Nominal pressure on the contact surface (Pa)
q	Specific power of friction ($W\;m^{-2}$)
$q_{0}$	Nominal value of the specific friction power ($W\;m^{-2}$)
t	Time (s)
$t_{s}$	Stop moment of the process (s)
T	Temperature (${}^{\circ }C$)
$T_{0}$	Initial temperature (${}^{\circ }C$)
$V_{0}$	Sliding velocity ($m\;s^{-1}$)
$Y_{n}(\cdot )$	Bessel functions of the *n*th order of the second kind
z	Spatial coordinate in axial direction (m)
$\gamma^{*}$	Gradient parameter of FGM
Λ	Temperature rise scaling factor (${}^{\circ }C$)
ε	Dimensionless coefficient of thermal activity
Θ	Temperature rise (${}^{\circ }C$)
$\Theta^{*}$	Dimensionless temperature rise
ρ	Density ($\mathrm{kg}\;m^{-3}$)
τ	Dimensionless time
$\tau_{s}$	Fourier number
ζ	Dimensionless spatial coordinate in axial direction
